# Impediment in upper airway stabilizing forces assessed by phrenic nerve stimulation in sleep apnea patients

**DOI:** 10.1186/1465-9921-6-99

**Published:** 2005-09-07

**Authors:** F Sériès, E Vérin, T Similowski

**Affiliations:** 1 Centre de recherche, Hôpital Laval, Institut universitaire de cardiologie et de pneumologie de l'Université Laval, Quebec City, Quebec, Canada; 2 UPRES EA 2397, Université Paris VI Pierre et Marie Curie, Paris, France; 3 Service de Physiologie, GRHV EA 3830, Université de Rouen, Rouen, France; 4 Service de Pneumologie, Groupe Hospitalier Pitié-Salpêtrière, Assistance Publique - Hôpitaux de Paris, Paris, France

**Keywords:** Sleep apnea, upper airway, phrenic stimulation

## Abstract

**Background:**

The forces developed during inspiration play a key role in determining upper airway stability and the occurrence of nocturnal breathing disorders. Phrenic nerve stimulation applied during wakefulness is a unique tool to assess Upper airway dynamic properties and to measure the overall mechanical effects of the inspiratory process on UA stability.

**Objectives:**

To compare the flow/pressure responses to inspiratory and expiratory twitches between sleep apnea subjects and normal subjects.

**Methods:**

Inspiratory and expiratory twitches using magnetic nerve stimulation completed in eleven untreated sleep apnea subjects and ten normal subjects.

**Results:**

In both groups, higher flow and pressure were reached during inspiratory twitches. The two groups showed no differences in expiratory twitch parameters. During inspiration, the pressure at which flow-limitation occurred was more negative in normals than in apneic subjects, but not reaching significance (p = 0.07). The relationship between pharyngeal pressure and flow adequately fitted with a polynomial regression model providing a measurement of upper airway critical pressure during twitch. This pressure significantly decreased in normals from expiratory to inspiratory twitches (-11.1 ± 1.6 and -15.7 ± 1.0 cm H_2_O respectively, 95% CI 1.6–7.6, p < 0.01), with no significant difference between the two measurements in apneic subjects. The inspiratory/expiratory difference in critical pressure was significantly correlated with the frequency of nocturnal breathing disorders.

**Conclusion:**

Inspiratory-related upper airway dilating forces are impeded in sleep apnea patients.

## Background

Sleep-related breathing disorders are fairly common in the general population [1]. In the majority of cases, they are obstructive in nature and are caused by recurrent sleep-related episodes of complete or partial upper airway (UA) closure. During these episodes, UA muscle activity progressively increases but the dilating force they develop is not sufficient to restore UA patency and normal ventilation until arousal or awakening. These breathing disturbances lead to intermittent hypoxia and hypercarbia, sleep disruption, and a sympathetic/parasympathetic imbalance that result in the development of daytime sleepiness [2], systemic hypertension [3], ischemic vascular disorders [4], and the clinical consequences of a prothrombotic state [5,6]. Given that the consequences of obstructive sleep apnea (OSA) are dramatically improved by the appropriate treatment [7-10] early diagnosis and treatment are essential.

The maintenance of UA patency depends on the balance between stabilizing and collapsing forces. The contraction of UA dilators generates the only stabilizing force that opposes a series of collapsing forces, including the effects of gravity-induced posterior displacement of UA structures, the negative inspiratory UA transmural pressure gradient, and surface tension forces. Numerous factors are involved in the determination of the effective stabilizing force applied to UA structures. These include the amount of UA neuromuscular activity [11], the physiological and histochemical properties of the muscles [12], the effectiveness of UA muscle contraction (i.e., synergistic or eccentric contractions, mechanical disadvantages) [13,14] and the mechanical coupling of UA muscles to surrounding soft tissues [15] Numerous attempts have been made to evaluate the mechanical importance of each of these individual factors, but the clinical and physiological impacts of combined UA stabilizing forces are unknown. A key observation is that UA compliance is increased in sleep apnea patients despite an higher neuromuscular drive to UA dilators in these patients during wakefulness and sleep [16,17], leading to the concept that the mechanical efficiency of UA dilating forces is impeded in these patients.

Phrenic nerve stimulation (PNS) can be used to study UA dynamics in conscious humans [18,19]. By provoking a diaphragm contraction independently of the neural activation of the UA dilators that normally precedes it, this technique mimics the dissociation between UA and diaphragm activities that is associated with the occurrence of OSA. Applying the PNS during expiration when UA dilators are only tonically active provides a pressure-flow relationship that reflects the mechanical properties of the tonically active UA. This pressure-flow relationship is dramatically modified if the PNS is applied when the UA dilators are phasically active [20]. Comparing the "expiratory" and "inspiratory" PNS-induced pressure-flow relationship provides a unique tool to measure the overall mechanical effects of the inspiratory process on UA stability. Since UA stability is vital in UA closure, the aim of the present work was to compare the ability of UA dilating forces to stabilize UA structures in normals and OSA subjects. Therefore, this work provides unique information on the impediment in upper airway stabilizing forces in sleep apnea patients.

## Materials and methods

### Patients

Twenty-one men (11 untreated sleep apneic, 10 normal) participated in this study. Normal subjects were recruited by newspaper advertisements and were free of symptoms suggestive of breathing disorders during sleep. The presence or absence of obstructive sleep apnea was always assessed with a polysomnographic recording. No subject was taking medication that could alter sleep or nocturnal breathing. The normal and OSA subjects had similar age and body mass index (BMI) ranges. The protocol was approved by the ethical review board of Hôpital Laval (Université Laval, Quebec City, Quebec, Canada). Subjects provided informed written consent.

### Sleep recordings

Polysomnographic recordings consisted of in-lab continuous acquisition of electroencephalogram, electroocculogram, submental electromyogram, arterial oxyhemoglobin saturation by transcutaneous pulsed oxymetry, naso-oral airflow with thermistors, nasal pressure with nasal cannula, chest and abdominal movements by impedance plethysmography (Respitrace™, Ambulatory Monitoring Inc., Ardsley, NY), electrocardiogram, and breath sounds. Sleep position was continuously assessed by the attending technician using an infrared camera. All variables were digitally recorded (Sandman Elite™ System, Mallinckrodt, Kenilworth, NJ).

### Phrenic nerve stimulation

#### Measurements

Surface recordings of right and left costal diaphragmatic EMG activities were obtained using silver cup electrodes placed on the axillary line of the sixth to eighth right and left intercostal spaces and connected to a electromyograph (Biopac System/Biopac, Santa Barbara, CA). A pressure-tipped catheter (Gaeltec, model CT/S X1058, Hackensack, NJ) was inserted in one nare after local anesthesia (1 ml of viscous 2% xylocaine) and located 16 cm from the nares to record hypophrayngeal (retroglossal) pressure (Pphar) [21]. An esophageal balloon was inserted through the other nare and located into the lower third of the oesophagus as assessed by the occlusion technique [22]. A plastic nasal stent (Nozovent; WPM International AB; Göteborg, Sweden) was placed in the anterior nares to prevent nasal collapse and the catheter was secured on the nose. A tightly fitting continuous positive pressure mask (Profile Light Nasal Mask, Respironics, Pittsburg, PA) was then placed over the nose. Airtightness of the mask was assessed by occluding the opening during maximal inspiratory efforts. A third catheter was passed through another opening of the mask to measure pressure inside the mask (Pmask). Flow was obtained from a pneumotachograph (Hans Rudolph, model 112467-3850A, Kansas City, MO) connected to the mask. Pressures and flow were digitally recorded at a 300 Hz sample rate (Digidata 1320, Axon Instruments, Foster City, CA). During the study, the subjects were seated in a comfortable armchair with a 60° inclination and their heads were maintained in a natural "neutral" position by a moulded pillow to ensure that the positions of the head and neck did not change during the experiment.

#### Phrenic nerve stimulation procedure

Bilateral anterior magnetic phrenic nerve stimulation (BAMPS) was performed using two Magstim 200 stimulators (Magstim Ltd,, Whitland, Dyfed, UK) connected to two 45 mm, figure eight-shaped coils with 90° handles, as previously described [20,23]. In brief, each stimulating coil was positioned antero-laterally over the anatomical landmark of the phrenic nerve in the neck at the posterior border of the strenomastoid muscle at the level of the cricoid cartilage. The handle of the coil was at a 45° angle to the mid-sagital and horizontal planes of the body. The optimal position and orientation of the coils was determined separately for each side at a 80 to 100 % stimulation intensity. A simplified recruitment curve (motor response to stimulation against stimulation intensity) was performed to verify the supramaximal nature of the stimulation. The two stimulators were triggered by a timer driven by the changes in flow direction. The 1 ms twitches were delivered after the operator-selected delay following inspiration or expiration onset had been reached.

### Protocol

All measurements were made with the subjects breathing exclusively by the nose. BAMPS was applied at end-expiration (2 s after expiratory onset) or early inspiration (200 ms after inspiratory onset) in random order. Considering that UA muscles activity reaches a plateau within 200 ms of inspiratory onset [24,25], this timing was selected for inspiratory twitch to restrict the influence of differences in lung volumes between inspiratory and expiratory twitches. Subjects were blind to the twitch timing. For each respiratory timing, one series of five stimulations was applied at each stimulation intensity between 70% and 100% of maximal stimulation intensity with a 10% stepwise increase in intensity.

## Data and statistical analyses

### Data Analysis

#### Polysomnographic studies

Sleep and respiratory variables were scored according to standard criteria [26]. Normal subjects were defined by an apnea + hypopnea index (AHI) ≤ 15/h.

#### Flow/pressure curve

**Figure 1:**
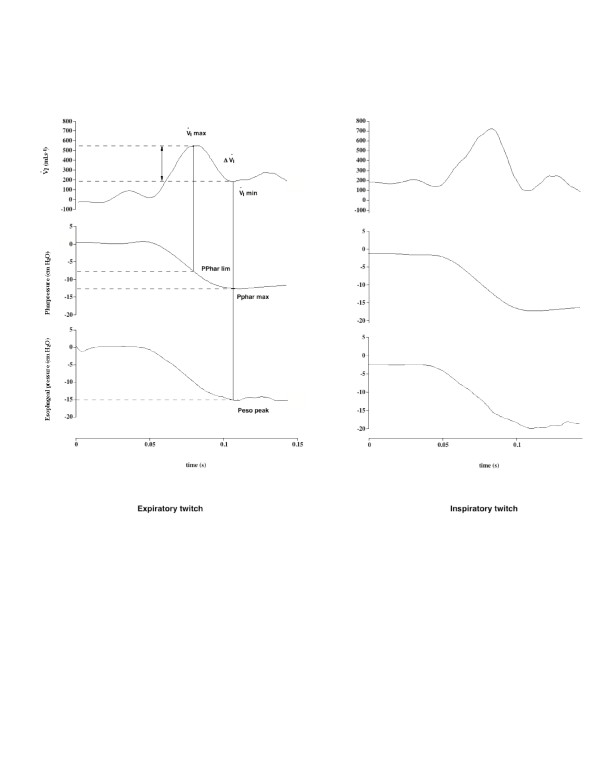
Example of the changes in inspiratory flow and pharyngeal andesophageal pressures in response to PNS-induced diaphragm twitch applied during expiration and inspiration in a representative normal subject. Time 0 indicates the application of the twitch. I max and I min stand respectively for the maximal and minimal flow values reached during the twitch. Pphar lim indicates the pharyngeal pressure value corresponding to Imax. Peso peak indicates the driving pressure corresponding to I min. See text for abbreviations.

Twitch-induced breaths were considered flow-limited when instantaneous flow plateaued or decreased despite a persistent increase in driving pressure. Representative tracings of the , Poeso and Pphar responses to twitch are presented in Figure 1. Flow increased in response to decreasing pharyngeal pressure up to a maximal flow value (Imax lim), which was reached at a pressure value corresponding to Pphar lim. For pressure values below Pphar lim, flow and pressure values were dissociated with a decrease in flow as pressure became more negative. ΔI represented the drop in  from Imax lim to flow nadir value (I min) for flow-limited twitches. The UA dynamic response of flow-limited twitches was characterized by modeling the twitch-related pressure-flow relationship from the rise in driving pressure up to its peak value as previously described [27]. According to this model, the pressure-flow relationship is fitted to a polynomial equation of the form  = k_1 _Pphar + k_2 _Pphar^2 ^using the least square method. Solving this equation for  = 0 with Pphar different from zero (Pphar = k_1_/k_2_) provides the pharyngeal pressure value at which the UA is totally closed. The k_1_/k_2 _ratio is equivalent to the UA critical pressure value during twitch and is thus a descriptor of UA stability (the higher the k_1_/k_2_, the higher the UA stability). Polynomial model fitting and determinations of k_1 _and k_2 _values were performed semi-automatically using custom-made software (Matlab, The Mathwork Inc., Boston, MA). Imax lim, Pphar lim, ΔI, and k_1_/k_2 _were considered to characterize FL twitches. The difference in the inspiratory and expiratory values of the k_1_/k_2 _ratio was considered as an index of UA aperture inspiratory efficiency, the more negative the index, the lesser the impediment of UA dilating forces. The following variables were also measured: (1) peak flow values (Imax) of non-flow limited twitches, (2) corresponding Pphar value, (3) peak Pphar and peak Peso (Pphar max and Peso max respectively), (4) Pphar at 400 ml/s, (5) ΔI, and (6) the % of twitches associated with flow limitation.

### Statistical Analysis

A nested split-plot design was first completed to separately analyze the effects of stimulation intensity and twitch respiratory timing on the measured variables in OSA and normals. Since stimulation intensity increased repeatedly for each subject, the respiratory timing factors were randomly assigned to the subjects (main plot) and the stimulation intensity factors were assigned to the split plot. This second factor was analyzed as a repeated-measures factor. A mixed model analysis was then performed with interaction terms between groups, stimulation intensity, and respiratory timing factors. A first-order autoregressive covariance structure was used to evaluate the influence of stimulation intensity and timing on the measured variables [28]. The heterogeneity in covariance structures was defined based on the effect between groups and the respiratory timing. The univariate normality assumptions were verified with Shapiro-Wilk tests, and multivariate normalities were verified with Mardia tests [29]. The results were considered significant with p values ≤ 0.05. All analyses were conducted using the SAS statistical package (SAS Institute Inc., Cary, NC).

## Results

No differences between normals and OSA subjects were found with regard to age, BMI, neck circumference, or pharyngeal resistance measured at iso-flow and peak flow (Table 1). In both groups, obstructive events represented 95 to 100 % of sleep-related breathing disturbances. Flow-limitation was consistently absent during tidal breathing in every subject. BAMPS-induced Peso max progressively rose with increasing stimulation intensity and was significantly higher during inspiratory than expiratory twitches (Table 2). For a given twitch timing, no significant difference was found between values obtained in normals and OSA subjects. The resulting flow was always higher when twitches were applied during inspiration than end-expiration with no difference between the two groups (Table 2), and remained unchanged with increasing stimulation intensity in both groups whatever the BAMPS timing.

**Table 1:** Anthropomorphic and polysomnographic characteristics of participating subjects. BMI: body mass index, NC: neck circumference, AHI: apnea + hypopnea index. Mean ± SEM.

	Normal Subjects (n = 10)	OSA Subjects (n = 11)	p
Age (y)	50 ± 1	50 ± 2	0.9
BMI (Kg.m^-2^)	27.2 ± 1.1	26.7 ± 1.2	0.8
NC (cm)	39.6 ± 0.9	40.5 ± 0.8	0.6
Pharyngeal resistance at 0.4 L.s^-1 ^(cm H_2_O.L^-1^.s)	3.9 ± 0.8	3.9 ± 0.7	0.3
Peak flow pharyngeal resistance (cm H_2_O.L^-1^.s)	4.4 ± 0.8	3.8 ± 0.7	0.2
AHI (n/h)	4.8 ± 1.0	29.4 ± 2.2	10^-4^

**Table 2:** Flow and pressure values obtained in response to 100% intensity PNS applied during expiration and inspiration in normals and OSA subjects. *: significant difference between inspiratory and expiratory values in a given group. Mean ± SEM.

	Expiratory		Inspiratory	
	Normal Subjects	OSA Subjects	Normal Subjects	OSA Subjects

_Imax _(ml.s^-1^)	716 ± 36	739 ± 51	990 ± 42 *	1038 ± 58 *
P_eso _peak at 100% stimulation I (cm H_2_O)	-17.6 ± 1.8	-14.0 ± 2.0	-21.1 ± 1.0 *	-17.7 ± 1.9 *
Pphar peak at 100% stimulation I (cm H_2_O)	-11.2 ± 2.2	-11.1 ± 2.1	-15.9 ± 1.5 *	-14.7 ± 1.4 *
R Phar 400 ml/s (cm H_2_O.ml^-1^.s)	9.0 ± 0.7	9.5 ± 0.9	10.5 ± 1.3	8.5 ± 0.9
R Phar _Imax _(cm H_2_O.ml^-1^.s)	9.2 ± 0.8	11.1 ± 1.3	10.2 ± 0.9	8.4 ± 0.7
_Imax _lim (ml.s^-1^)	742 ± 248	752 ± 352	981 ± 297 *	1014 ± 348 *
Pphar lim (cm H_2_O)	-8.4 ± 4.2	-7.2 ± 3.3	-12.5 ± 4.1 *	-9.6 ± 3.6 *
Δ_I _(ml.s^-1^)	234 ± 26	256 ± 37	258 ± 35	234 ± 24
% twitches with FL	76.6 ± 3.4	79.0 ± 4.4	75.0 ± 3.5	84.4 ± 4.1

### Twitch-induced flow-limited responses

**Figure 2:**
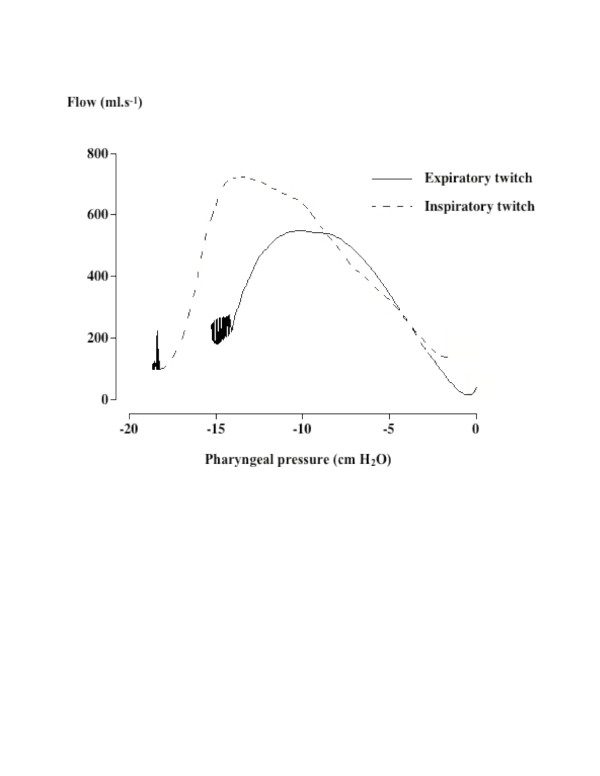
Plot of the flow/pharyngeal pressure relationships obtained with data presented in Figure 1. The flow-limited nature of the twitch-induced flow is clearly demonstrated by the flow drop associated with the pharyngeal pressure decrease once Pphar lim and the corresponding I max have been reached. A clear difference in the flow/pressure curves can be seen between the expiratory and inspiratory twitches.

Figures 1 and 2 depict expiratory and inspiratory twitch-induced instantaneous flow and pressure responses and the corresponding pressure/flow curves. The flow-limited nature of the twitch flow is clearly demonstrated by the flow/pressure pattern. Despite a progressive decrease in pharyngeal pressure, a rise in flow was only observed up to a pharyngeal pressure threshold before plateauing occurred and instantaneous flow dropped. The percentage of twitches with a flow-limitation pattern was identical in the two groups for expiratory twitches. The percentage of flow-limited twitches did not differ between expiratory and inspiratory twitches (Table 2). In both groups, _Imax _lim was significantly higher for inspiratory than expiratory twitches (Table 2), with no influence of stimulation intensity. For a given timing, no difference was found in _Imax _between normal subjects and OSA. Pphar lim progressively increased with rising stimulation intensity for both inspiratory and expiratory twitches. In both groups, Pphar lim was significantly lower during inspiratory twitches. Pphar lim measured during inspiratory twitches were more negative in normals than in OSA but the difference between the two groups did not reach significance (p = 0.07) (Table 2). P phar peak of flow-limited twitches also decreased with increasing stimulation intensity and reached more negative values during inspiratory twitches. No difference was found in the Δ_I _between the two groups for each PNS timing (Table 2).

### Driving pressure-flow relationship

**Figure 3:**
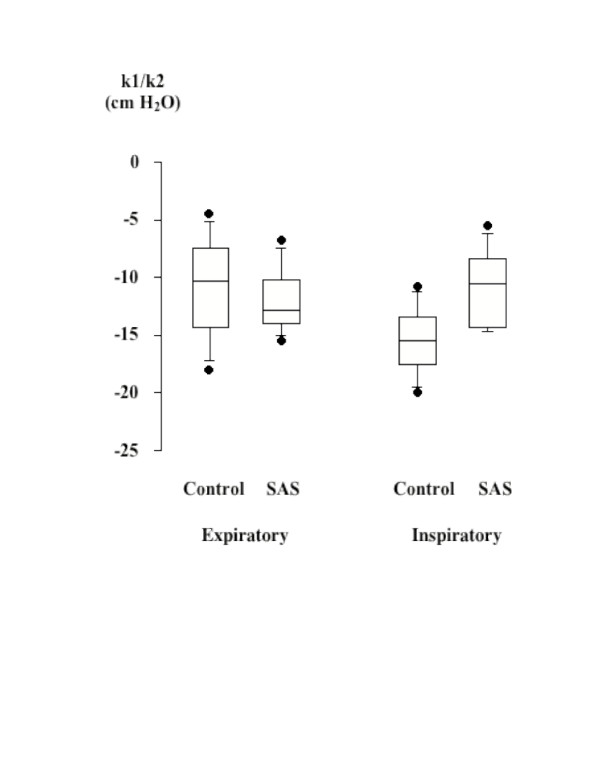
k1/k2 ratios for expiratory and inspiratory twitches in the normal and OSA groups. There was no difference between the two groups for expiratory twitches while the ratio decreased significantly during inspiratory twitches in normals and increased in OSA subjects. Boxes identify the 25 to 75 th percentiles of the data with the median value indicated. Horizontal lines outside the boxes depict the 10 to 90 th percentiles. Closed circles represent outliers. *: significant difference between inspiratory and expiratory values.

**Figure 4:**
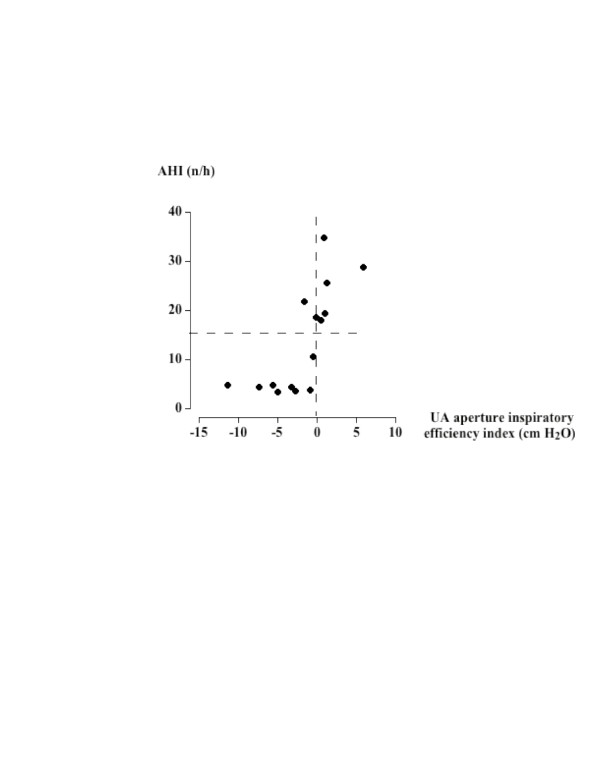
Correlation between individual values of the apnea + hypopnea index and the corresponding UA aperture inspiratory efficiency index.

The  = k_1_Pphar+k_2_Pphar^2 ^model adequately described the relationship between pharyngeal pressure and the related flow of flow-limited twitches in all but one subject (r 0.71 to 1.0, mean r: 0.91, p < 0.0001). The model could not be applied on flow-limited twitches in two OSA subjects due to the presence of artefacts. In the other subjects, artefacts occurred in 13.6 % of twitches that prevented data interpretation at some stimulation intensities between 60 to 90%. The index of UA aperture inspiratory efficiency was significantly influenced by stimulation intensity, twitch timing, and subject group. It decreased with rising stimulation intensity, and was lower during inspiratory than expiratory twitches (Figure 3). In normals, k_1_/k_2 _significantly decreased from expiratory to inspiratory twitches (-11.1 ± 1.6 and -15.7 ± 1.0 cm H_2_O respectively, 95% CI 1.6–7.6, p < 0.01), with no difference between the two twitches timings in OSA subjects (-12.0 ± 1.1 and -10.9 ± 1.3 cm H_2_O respectively, 95% CI -3.2 to 1.1, p = 0.3). Similar results were obtained when only considering data collected at 100 % stimulation intensity. The UA aperture inspiratory efficiency was higher in normals than in OSA subjects. The individual values of this index measured at 100% stimulation intensity positively correlated with the apnea + hypopnea index (r = 0.73, p = 6.10^-4^, Figure 4).

## Discussion

This study showed that inspiratory UA stabilizing forces were altered in OSA subjects compared to normals matched for age, sex, and BMI. The magnitude of the decrease was statistically associated with the frequency of nocturnal obstructive events.

The assessment of UA stability is a complex issue because of its dynamic nature. The role of UA dilating forces in the pathophysiology of UA closure has thus to be considered from a mechanistic point of view that takes into account the entire the sequence of events from the rise in respiratory and UA muscle activity to UA tissue displacement and/or stretching. UA shape and dimension determine the load imposed on phasic stabilizing forces by influencing the amount of tissue displacement resulting from a given increase in dilator activation [30]. As for the dynamic properties of UA during inspiratory twitches, the synergy between agonistic/antagonist muscle activation as well as the interaction with tracheal traction are important determinants of the UA stabilization process [11,13,31-33]. Another important aspect is the occurrence of eccentric contractions due to the drop in transmural pressure gradient [14]. Such contraction pattern has been found to contribute to the development of muscle fatigue in peripheral skeletal muscles [34]. Lastly, the stretching effect of UA tissues during inspiration largely depends on the mechanical properties of the soft tissues surrounding the UA muscles [15]. In this context, the PNS technique applied during expiration or inspiration provides unique information in that it can be used to differentiate UA tonic or phasic mechanical properties. The UA aperture inspiratory efficiency index thus reflects the overall effect of inspiratory UA traction forces and takes into account the efficiency of the contraction of UA muscles to generate tension, the transmission of this tension to surrounding tissues, as well as the effects of inspiratory tracheal traction.

Characterizing the entire pressure-flow response instead of specific flow and pressure values to evaluate twitch-induced UA dynamic improves the sensitivity of this method for detecting changes in UA properties. This is supported by the non-significant differences in I and Pphar measurements observed between the two groups. Indeed, the twitch-induced flow-pressure curve has an M-shape due to the initial rise in inspiratory flow up to a maximal value in response to driving pressure before decreasing to a minimal value that usually corresponds to the peak driving pressure, and then increasing again. This late increase in flow may be caused by a negative pressure-triggered reflex activation of UA dilators. The first part of the flow-pressure relationship was adequately described by a polynomial regression model I = k_1_Pphar + k_2 _Pphar^2 ^with a negative k_2 _value. The k_1 _and k_2 _coefficients relate to airway conductance, k_1 _being the counterpart of RI max and k_2 _that of RI min. The k_1_/k_2 _ratio determines the driving pressure (in this case pharyngeal pressure) that should lead to the complete collapse of the UA and therefore is an index of UA stability in response to an over-imposed negative intra-thoracic pressure pulse. The fact that k_1_/k_2 _decreases from expiration to inspiration in normals indicates that phasic activity of UA dilators stabilizes the UA.

It is interesting to note that the difference in UA stability between normals and OSA subjects was influenced by the respiratory time during which the PNS was applied. During expiration, there was no difference in critical pressure between the two groups. This differs from the results previously published by our laboratory [35]. However, such apparent discrepancies can be easily accounted for by (1) the anthropometric differences between the control and apneic groups in our previous study possibly originating from the patients recruitment procedure, (2) the completion of twitches exclusively at 100 % intensity in our previous study and (3) the differences in driving pressure assessment (esophageal pressure vs. pharyngeal pressure). The absence of difference in UA mechanics in non-phasically active UA could reflect the mechanical effect of higher UA muscle tonic activity in sleep apnea patients [36] to compensate for the deleterious effect of the alteration in UA shape and/or dimension. Moving from expiration to inspiration was associated with an increase in critical pressure in normals and with a paradoxical decrease in critical pressure in OSA subjects.

Before elaborating on the possible explanation for such findings, it is first important to consider that in our study, UA stability was assessed by the flow/pressure response to an over-imposed negative intra-thoracic pressure pulse. The amplitude of this one can be considered as in the physiological range since it is generated by the respiratory system following maximal stimulation of the diaphragm via the phrenic nerves. However, the steepness of the rise in driving pressure that is specific to the phrenic nerve stimulation response (square pressure wave) and differs from the pressure profile generated during tidal breathing.

Several factors could account for the inability of UA dilating forces to overcome a super-imposed collapsing force during inspiration in OSA subjects such as a smaller increase in inspiratory EMG activity, an altered EMG response to negative pressure, or altered mechanical efficiency of UA dilator contraction. The phasic activity of UA muscles is known to preceed that of respiratory muscles in normals [37,38] as well as in OSA subjects while awake and during the post-apneic period [39]. Therefore, it is reasonable to assume that UA muscle recruitment was maximal (or almost maximal) at the time of application of inspiratory BAMPS in both groups. Furthermore, previous results suggest that this phasic activity should represent a higher percentage of spontaneous maximal UA neuromuscular activity in OSA subjects than in normals [16]. Concerning the reflex-induced rise in UA muscles activity following twitches, this one should be larger for inspiratory than for expiratory twitches [40], with a greater response amplitude in OSA subjects than normals [41]. Should this reflex response have influenced the twitch-induced flow/pressure response, it would attenuate the difference in UA dynamics that we observed between normals and OSA. In this context, the inspiratory/expiratory difference in UA stability measured with our technique reflected the net effect of UA dilating forces on UA stability. Our results thus support the concept that the splinting effects of inspiratory forces is altered in OSA subjects as a consequence of the circumstances described above and contribute to a decrease in the stretching effect of UA muscle contraction. Given the dramatic impact of sleep on upper airway muscles' responses to negative pressure, it can be speculated that the observed difference in UA aperture inspiratory efficiency between apneic and non-apneic subjects should be larger during sleep.

We found a significant relationship between AHI and the UA aperture inspiratory efficiency index. Even if there is no causal link between these parameters, the rise in AHI with decreasing the UA aperture inspiratory efficiency suggests that net inspiratory dilating forces are involved in UA stability and nocturnal obstructive breathing disorders. Given the importance of weight in determining the frequency of nocturnal breathing disorders and UA collapsibility [42], it should be remembered that our normal and apneic groups had identical anthropomorphic characteristics. Our results thus confirm the concept of the role of UA collapsibility in the development of obstructive breathing disorders [43-45], and also indicate that inspiratory stabilizing forces, as assessed in the present study, are cornerstones in the occurrence of such disorders. This may explain the conflicting results reported in the literature on the relationship between AHI and critical pressure when the relationship is assessed using different techniques.

We feel that the present study provides a useful contribution to the understanding of the effects of inspiratory stabilizing forces on the behaviour of the UA and its role in the pathophysiology of sleep apnea syndrome.

## Competing interests

The author(s) declare that they have no financial or non-financial competing interests

## Authors' contributions

FS conceived of the study, elaborated its design and contributed to its coordination. TS and EV participated in the revision of the design of the study. EV performed the analysis of the flow/pressure curves. All authors participated in and helped to draft the manuscript. All authors read and approved the final manuscript.
